# *χ*^(2)^ nonlinear photonics in integrated microresonators

**DOI:** 10.1007/s12200-023-00073-4

**Published:** 2023-07-17

**Authors:** Pengfei Liu, Hao Wen, Linhao Ren, Lei Shi, Xinliang Zhang

**Affiliations:** 1 Wuhan National Laboratory for Optoelectronics, Huazhong University of Science and Technology, Wuhan, 430074 China; 2 Optics Valley Laboratory, Wuhan, 430074 China

**Keywords:** Second-order nonlinearity, Integrated microresonators, Frequency conversion, Electro-optic effect

## Abstract

Second-order (*χ*^(2)^) optical nonlinearity is one of the most common mechanisms for modulating and generating coherent light in photonic devices. Due to strong photon confinement and long photon lifetime, integrated microresonators have emerged as an ideal platform for investigation of nonlinear optical effects. However, existing silicon-based materials lack a *χ*^(2)^ response due to their centrosymmetric structures. A variety of novel material platforms possessing *χ*^(2)^ nonlinearity have been developed over the past two decades. This review comprehensively summarizes the progress of second-order nonlinear optical effects in integrated microresonators. First, the basic principles of *χ*^(2)^ nonlinear effects are introduced. Afterward, we highlight the commonly used *χ*^(2)^ nonlinear optical materials, including their material properties and respective functional devices. We also discuss the prospects and challenges of utilizing *χ*^(2)^ nonlinearity in the field of integrated microcavity photonics.

**Graphical Abstract:**

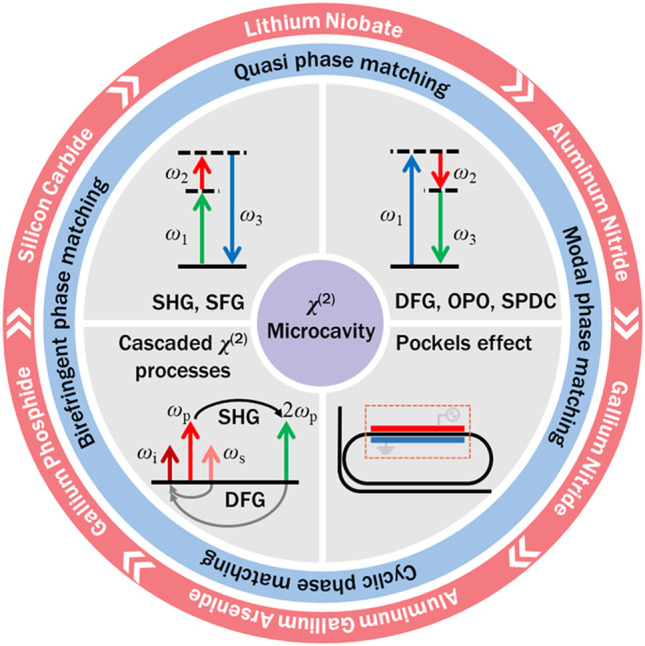

## Introduction

Since the first experimental demonstration of second-harmonic generation (SHG) in 1961 [1], nonlinear optics has evolved into an exceptionally active research area. Remarkably, *χ*^(2)^ nonlinear optical processes are the basis of many classical and quantum applications [2–6]. For example, coherent frequency conversion enables the generation of new optical frequencies; this capability provides a viable solution for the realization of new laser sources. In quantum optics, photon-pair sources based on spontaneous parametric down-conversion (SPDC) have enabled considerable advances in quantum communication and computation [7–10]. In addition, the high-speed electro-optic (EO) modulator is the key component for long-haul communication systems [11]. Indeed, the exploration of *χ*^(2)^ nonlinearity has been commercialized based on nonlinear crystals such as potassium titanyl phosphate (KTP) and lithium niobate (LiNbO_3_). However, these bulk devices have large footprints and high costs, and these issues ultimately limit their applications as compact and scalable components. Optical microresonators provide an ideal platform for realizing efficient nonlinear effects at the micro- and nano-scales [12–15]. One of the most widely used resonators is the whispering-gallery-mode resonator (WGMR). Due to the circular edge of the WGMR, light can be confined within a small volume through total internal reflection. The resulting high optical field intensity and ultra-long photon lifetime strongly enhance the light-matter interactions, allowing manipulation of nonlinear optical processes under low-power continuous-wave pumping. In addition, millimeter-size resonators can be integrated into chip-scale nonlinear photonic devices [16]. Combined with these intriguing properties, WGMRs allow not only exploration of nonlinear interactions at the single-photon level, but also translation of laboratory demonstrations into practical applications.

Integrated microresonators have attracted considerable attention due to their low footprint, high integration density and low power consumption [17–20]. The material platforms for on-chip microresonators are crucial for practical applications. Silicon photonics is the widely recognized basis for photonic integrated circuits (PICs) owing to the compatibility with the complementary metal oxide semiconductor (CMOS) processes [21–24]. However, the commonly employed materials in silicon-based photonics, including silicon (Si), silicon nitride (Si_3_N_4_) and silicon dioxide (SiO_2_), do not possess *χ*^(2)^ nonlinearity due to their centrosymmetric crystalline structures [25, 26]. Despite photo-induced SHG having recently been demonstrated in Si_3_N_4_ microresonators, their effective *χ*^(2)^ are two orders of magnitude lower than those in typical *χ*^(2)^ nonlinear optical materials [27, 28]. Therefore, exploration of new materials with ultralow loss and strong *χ*^(2)^ nonlinearity is highly demanded. Thus far, lithium niobate (LiNbO_3_, abbreviated to LN) [29–32], silicon carbide (SiC) [33], and III–V semiconducting compounds including aluminum nitride (AlN) [34, 35], gallium nitride (GaN) [36], gallium phosphide (GaP) [37], and aluminum gallium arsenide (AlGaAs) [38], are typical materials for investigating *χ*^(2)^ nonlinear effects. Table 1 lists key optical properties of these materials and typical quality factors (*Q*-factors) of the corresponding integrated microresonators. Excellent nonlinear optical properties of LN, especially the electro-optic behavior, can enable ultrafast light modulation and efficient frequency conversion. SiC as a CMOS-compatible material offers unique properties for quantum and nonlinear optical applications. III-N materials possess large bandgaps, therefore they can enable optical devices working in the ultraviolet (UV) to visible wavelength range. GaP has a high refractive index, enabling good light field confinement and implying a large *χ*^(2)^ nonlinearity coefficient. Last but not least, AlGaAs exhibits both optical gain and strong optical nonlinearities, and has been used in a wide range of on-chip nonlinear photonic devices. Despite the different material properties, the *Q*-factors of integrated *χ*^(2)^ microresonators can reach above 10^7^, offering excellent platforms that achieve nonlinear optical modulation and frequency conversion.

**Table 1:** Key optical properties of typical *χ*^(2)^ nonlinear materials for integrated microcavity photonics

Material	Refractive index	Bandgap/eV	Transparency window/μm	Electro-optic coefficient/(pm⋅V^−1^)	Second-order nonlinearity/(pm⋅V^−1^)	Quality factor/million
LN	*n*_o_ = 2.21 *n*_e_ = 2.14	3.9	0.4–5	*r*_33_ = 31 @ 633 nm [39]	*d*_33_ = − 27 @ 1064 nm [39]	120 [40]
AlN	*n*_o_ = 2.12 *n*_e_ = 2.16	6.2	0.2–13.6	*r*_33_ = 1 @ 633 nm [41]	*d*_33_ = 4.7 @ 1520 nm [42]	3.7 [43]
SiC	*n*_o_ = 2.56 *n*_e_ = 2.60	2.3–3.2	0.37–5.6	*r*_33_ = 1.5 @ 1550 nm [44]	*d*_33_ = 30 @ 1550 nm [45]	7 [46]
GaN	*n*_o_ = 2.32 *n*_e_ = 2.30	3.4	0.365–13.6	*r*_33_ = 1.91 @ 633 nm [47]	*d*_36_ = − 4.6 @ 1030 nm [48]	2.5 [36]
GaP	3.05	2.26	0.5–11	*r*_41_ = 1.1 @ 1153 nm [49]	*d*_36_ = 159 @ 852 nm [50]	0.25 [37]
AlGaAs	3.4	1.42	0.87–19	*r*_41_ = 1.5 @ 1520 nm [51]	*d*_36_ = 119 @ 1533 nm [50]	6 [52]

In this paper, we review the research progress over the past two decades of second-order nonlinear optical effects in integrated microresonators. First, we introduce the fundamental principles of the EO effect and the three-wave mixing process, as well as the methods for achieving phase matching. Then we summarize typical *χ*^(2)^ nonlinear materials for integrated photonics. For each of these materials, a brief introduction to the basic optical properties and the research progress of *χ*^(2)^ nonlinear effects is presented. Finally, a short summary and an outlook are given to indicate some possible topics of future research in this field.

## Theoretical basis

### Basic theory of *χ*^(2)^ nonlinear effects

Second-order optical nonlinearity originates from the quadratic response of material polarization to external electric field [53]:

1 $${\varvec{P}}^{(2)} (t) = \varepsilon_{0} \chi^{(2)} {\varvec{E}}^{2} (t),$$

where *ε*_0_ and *χ*^(2)^ are the free-space permittivity and the second-order nonlinear susceptibility, respectively. In the presence of a strong pump light, the nonlinear response can cross-link different frequency components, causing modulations, conversions and oscillations within the spectrum. These parametric processes provide commonly used methods for generating light at new frequencies [54].

The linear EO effect, also known as the Pockels effect, is one of the most fundamental nonlinear effects. As a second-order nonlinearity, it describes the interaction between an optical field and a static electric field, and this interaction can change the refractive index linearly [55]:

2 $$n({\varvec{E}}) = n - \frac{1}{2}n^{3} {\varvec{rE}}.$$

The quantity ***r*** is the electro-optic coefficient that gives the rate at which the refractive index changes with the applied electrical field. The Pockels effect is the underlying principle for realizing EO modulation and EO comb generation. Particularly in integrated microresonators, this effect leads to a shift of the resonance frequency for matching with other embedded devices [56].

Now, we turn to discuss second-order interactions of optical fields, i.e., three-wave mixing. Considering light fields at two frequencies *ω*_1_ and *ω*_2_ existing in a *χ*^(2)^ nonlinear material:

3 $${\varvec{E}}(t) = {\varvec{E}}_{1} {\text{e}}^{{ - {\text{i}}\omega_{1} t}} + {\varvec{E}}_{2} {\text{e}}^{{ - {\text{i}}\omega_{2} t}} + c.c.$$

Substituting this expression into Eq. (1), we get the second-order nonlinear polarization:

4 $$\begin{aligned} {\varvec{P}}^{(2)} (t) & = \varepsilon_{0} \chi^{(2)} \left[ {{\varvec{E}}_{1}^{2} {\text{e}}^{{ - {\text{2i}}\omega_{1} t}} + {\varvec{E}}_{2}^{2} {\text{e}}^{{ - 2{\text{i}}\omega_{2} t}} + 2{\varvec{E}}_{1} {\varvec{E}}_{2} {\text{e}}^{{ - {\text{i}}(\omega_{1} + \omega_{2} )t}} + 2{\varvec{E}}_{1} {\varvec{E}}_{2}^{ * } {\text{e}}^{{ - {\text{i}}(\omega_{1} - \omega_{2} )t}} + c.c.} \right] \\ & \quad + 2\varepsilon_{0} \chi^{(2)} ({\varvec{E}}_{1} {\varvec{E}}_{1}^{ * } + {\varvec{E}}_{2} {\varvec{E}}_{2}^{ * } ). \\ \end{aligned}$$

According to Eq. (4), new frequency components other than *ω*_1_ and *ω*_2_ appear, corresponding to different nonlinear frequency conversion processes. The first two terms give rise to SHG, where the pump frequency is doubled through the light-matter interaction. The next two terms give rise to sum and difference frequency generation (SFG and DFG), where two pump lights with frequency *ω*_1_ and *ω*_2_ meet inside the nonlinear medium to generate the third frequency, which can be a sum or difference of the two pump frequencies, i.e., *ω*_1_ ± *ω*_2_ → *ω*_3_. There is a relevant process, optical parametric oscillation (OPO), where a strong pump at a short wavelength generates idler and signal lights, satisfying *ω*_3_ → *ω*_1_ + *ω*_2_. The generated idler and signal lights can further interact with the pump light through DFG to form an oscillation.

It is well-known that energy and momentum conservations are critical for nonlinear optical processes. Here, we consider SHG in a microresonator as an example, where two photons at the pump mode (*a*) are converted into one photon at the second-harmonic mode (*b*). This process can be described by the Hamiltonian

5 $$H = \omega_{a} \hat{a}^{\dag } \hat{a} + \omega_{b} \hat{b}^{\dag } \hat{b} + g\left[ {(\hat{a}^{\dag } )^{2} \hat{b} + \hat{a}^{2} \hat{b}^{\dag } } \right] + \varepsilon_{{\text{p}}} (\hat{a}{\text{e}}^{{{\text{i}}\omega_{{\text{p}}} t}} + \hat{a}^{\dag } {\text{e}}^{{ - {\text{i}}\omega_{{\text{p}}} t}} ),$$

where $\hat{a}{(}\hat{a}^{\dag } )$ and $\hat{b} \left( {\hat{b}^{\dag } } \right)$ are the annihilation (creation) operators for the pump and SHG mode, respectively; $\varepsilon_{{\text{p}}} = \sqrt {2\kappa_{{a,{\text{c}}}} P_{{\text{p}}} /\hbar \omega_{{\text{p}}} }$ is the pump field strength for a pump light at the frequency *ω*_p_ and power *P*_p_, and *κ*_*a*,c_ is the external coupling rate of mode *a*. The nonlinear coupling strength *g* is given by [57]

6 $$g = \zeta \sqrt {\frac{{\hbar \omega_{a}^{2} \omega_{b} }}{{\varepsilon_{0} 2{\uppi }R}}} \frac{1}{{\varepsilon_{a} \sqrt {\varepsilon_{b} } }}\frac{{3\chi^{(2)} }}{4\sqrt 2 }\delta (m_{b} - 2m_{a} ),$$

where *R* is the radius of the microresonator; *ε*_*a*(*b*)_ is the relative permittivity; *m*_*a*(*b*)_ is the azimuthal mode number and *ζ* is the mode overlap factor. Additionally, the Kronecker delta function indicates that *g* is nonzero only when *m*_*b*_ − 2*m*_*a*_ = 0, which corresponds to the phase matching condition. Under the non-depletion approximation, the SHG conversion efficiency reads

7 $$\eta = g^{2} \frac{{2\kappa_{{b,{\text{c}}}} }}{{\delta_{b}^{2} + \left( {\kappa_{b,0} + \kappa_{{b,{\text{c}}}} } \right)^{2} }}\left( {\frac{{2\kappa_{{a,{\text{c}}}} }}{{\delta_{a}^{2} + \left( {\kappa_{a,0} + \kappa_{{a,{\text{c}}}} } \right)^{2} }}} \right)\frac{{\hbar \omega_{b} }}{{\left( {\hbar \omega_{{\text{p}}} } \right)^{2} }},$$

where *δ*_*a*(*b*)_ is the frequency detuning for mode *a* (*b*); *κ*_*a*(*b*),0_ and *κ*_*a*(*b*),c_ are the intrinsic loss rate and coupling rate, respectively.

Theoretical analyses of other *χ*^(2)^ nonlinear processes are similar to those of SHG. According to Eqs. (6) and (7), an efficient nonlinear process requires: (1) high nonlinear coupling strength, corresponding to large *χ*^(2)^, large mode overlap as well as small microresonator radius; (2) fulfillment of the phase matching condition, i.e., Δ*m* = 0; (3) high intrinsic quality factors for all involved modes; (4) operating near the critical coupling condition.

### Phase matching

As mentioned in Sect. 2.1, phase matching requires Δ*m* = 0. Several methods have been developed for achieving phase matching in optical microresonators. They can be divided into two broad categories [58]: perfect phase matching and quasi-phase matching (QPM).

**Fig. 1:**
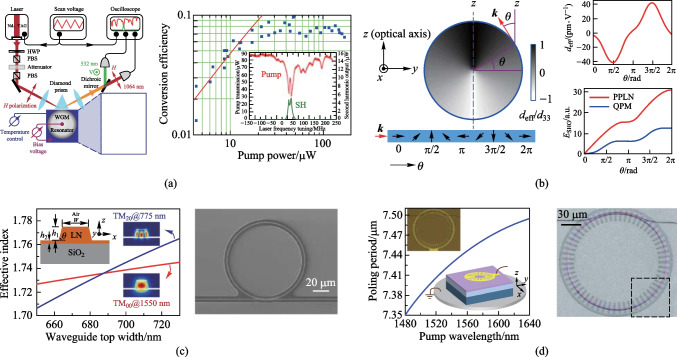
Phase-matching methods used for optical microresonators. **a** Birefringent phase matching. **b** Cyclic phase matching. **c** Modal phase matching. **d** Periodic poling in an LN microresonator. **a** Reprinted with permission from Ref. [59]. Copyright 2010, American Physical Society. **b** Reprinted with permission from Ref. [63]. Copyright 2019, American Physical Society. **c** Reprinted with permission from Ref. [66]. Copyright 2019, American Physical Society. **d** Reprinted with permission from Ref. [70]. Copyright 2019, Optica

Birefringent phase matching (BPM) is commonly used for achieving perfect phase matching in anisotropic materials. Pump and signal lights can be polarized along different axes to counteract the refractive index difference at different frequencies. The configuration, in which the polarization of the low-frequency fundamental mode is perpendicular to that of the high-frequency mode, is known as type-I phase matching. In contrast, the configuration in which two fundamental modes have orthogonal polarizations, is known as type-II phase matching. In 2010, Fürst et al. implemented efficient SHG using BPM in a MgO-doped LN disk resonator [59]. As shown in Fig. 1a, the birefringent indices can be dynamically tuned by adjusting the temperature and the applied bias voltage. Although this method enables a high conversion efficiency at a low pump power, BPM is rarely used in microresonators due to the spatial walk-off issue and the spectral range limitation.

Cyclic phase matching (CPM) is often used in microrings or microdisks where the optical axis is in the plane of the resonator [60, 61]. For example, on an X/Y-cut LN thin film, the polarization of transverse magnetic (TM) modes is perpendicular to the optical axis, resulting in a constant ordinary refractive index. Transverse electric (TE) modes, on the other hand, experience refractive index that oscillates between the ordinary and extraordinary values as light propagates in a microresonator. Thus, type-I phase matching can be achieved at four azimuthal angles within one round trip. This method enables a much wider phase matching bandwidth at the price of a reduced conversion efficiency [62]. Similar to CPM, natural QPM was proposed to achieve type-0 phase matching in the above structures. Here, type-0 phase matching configuration refers to the case in which the polarizations direction of the three involved modes are the same and along the extraordinary axis. Figure 1b shows an example, with both the nonlinear coefficient and the refractive index of TE modes oscillating along the periphery of a microresonator [63]. This is analogous to the periodic domain inversion, as will be discussed later. Using this method, broadband SHG with an ultrahigh conversion efficiency of ~ 470,000%/W was reported in an LN microdisk [40].

Modal phase matching (MPM) is particularly important in microresonators, since it is not possible to rotate the optical axis once the devices have been fabricated. In WGMRs, the effective refractive index of a cavity mode depends on the wavelength as well as the geometrical structure of the waveguides. For a given multimode waveguide, dispersion engineering can be employed to compensate for the material dispersion at different wavelengths [42, 64, 65]. As depicted in Fig. 1c, this effective index match is achieved for a 775-nm TM_20_ mode and a 1550-nm TM_00_ mode by varying the waveguide width of an LN microring [66]. Despite MPM offering a simpler fabrication process, the high-order modes have weaker field confinement and less overlap integral with the fundamental mode. This results in a higher propagation loss and limited conversion efficiency.

QPM requires periodical inversion of the nonlinear susceptibility to ensure efficient power transferring from the pump mode to the targeted mode [67–69]. This can be achieved in ferroelectric crystals, such as LN, where the sign of *χ*^(2)^ can be inverted by applying a high electric field. Such a process is called periodic poling, and the optimum poling period is Λ = 2π/Δ*k.* One can think that the poled structure imparts an additional wavevector *K* = 2π/Λ, to compensate the intrinsic wavevector mismatch Δ*k*. Figure 1d shows an example of periodic poling in an LN microring [70]. The radial electrodes are first patterned on the top of the microresonator, then the domain inversion is enabled by keeping the bottom plate as the electrical ground and applying a series of short pulses at an elevated temperature. In addition, different nondestructive techniques are used to visualize the poled domains, such as piezo-response force microscopy (PFM) [71], selective etching [72], and second-harmonic confocal microscopy [73]. Based on QPM, efficient classical and quantum frequency conversions have been demonstrated in microresonators [74–76]. It should be noted QPM is currently widely used in LN, as electric-field poling and QPM engineering capability are not available in most other *χ*^(2)^ nonlinear materials, which lack the necessary ferroelectricities [77, 78].

## Material platforms

### LN

LN is one of the most versatile and attractive photonic material platforms, due to its outstanding optical properties, such as strong electro-optic, nonlinear-optic, and acousto-optic effects, as well as its large refractive index (*n*_o_ = 2.21 and *n*_e_ = 2.14 at 1550 nm), wide transparency window (from 400 nm to 5 μm) and relatively stable physical and chemical characteristics [79–82]. Along with the rapid development of fabrication techniques, “Smart-Cut” technology that is commonly used on the silicon-on-insulator (SOI) platform has become the standard fabrication process for manufacture of high-quality thin films of LN-on-insulator (LNOI) [83–85]. Compared with traditional LN waveguides, thin-film LNOI (TFLNOI) waveguides possess a higher refractive index contrast (~ 0.7) and more flexibility in the fabrication process. In addition, low-loss waveguides on TFLNOI (as low as 0.027 dB/cm) have been developed [86]. Even at the wafer scale, a propagation loss of 0.27 dB/cm has also been demonstrated [87]. Following the maturity of fabrication techniques of TFLNOI waveguides, the fabrication of high-*Q* microresonators has been achieved by dry etching (highest *Q*-factor ~ 10^7^) [88–92], wet etching (*Q*-factor ~ 10^6^) [93], femtosecond laser direct writing followed by chemical mechanical polishing (highest *Q*-factor ~ 10^8^) [40, 94–96], direct focused ion beam (FIB) milling (*Q*-factor ~ 10^5^) [97], or femtosecond laser ablation followed by FIB sidewall milling and high-temperature annealing (highest *Q*-factor ~ 10^7^) [60, 63, 98, 99]. On-chip microresonators with high *Q*-factors, such as microrings, microdisks, micro-racetracks, and photonic crystal (PhC) cavities, have enabled numerous second-order nonlinear optical studies based on TFLNOI [100–104], and have facilitated applications in optical communications [105–111], quantum photonics [112–116], spectroscopy [117], and so on.

SHG is considered the most straightforward and the earliest nonlinear application of microresonators on TFLNOI. In 2014, Wang et al. demonstrated a microdisk on TFLNOI, and a conversion efficiency of 10.9%/W was achieved [118]. In 2015, by using a microdisk on Z-cut LN thin film featuring a *Q*-factor of 2.45 × 10^6^ at 1550 nm, the normalized conversion efficiency was measured to be 1.35%/W under CW pumping [119]. To realize a high conversion efficiency, the phase matching condition has to be perfectly fulfilled; Lin et al. made use of an X-cut microdisk to obtain a conversion efficiency of 110.6%/W via CPM [60]. Chen et al. achieved a conversion efficiency of 260%/W based on a Z-cut microring by utilizing the MPM mechanism [65]. In 2019, Luo et al. demonstrated SHG in a Z-cut microring with a loaded *Q*-factor around 10^5^ in the 1500- and 780-nm bands, realizing a conversion efficiency of 1500%/W through MPM [66].

**Fig. 2:**
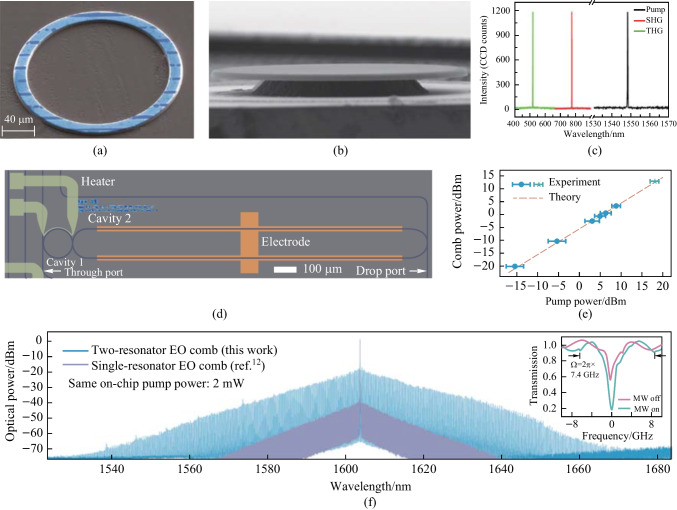
**a** False-color SEM image of a PPLN microring with a diameter of 216 μm. **b** SEM image of a natural QPM microdisk. **c** Spectra of the pump light, second-harmonic wave and third-harmonic wave. **d** EO comb generator based on coupled microresonators. **e** Measured comb power versus the pump power, exhibiting a high conversion efficiency of 30%. **f** Spectra of the coupled-resonator EO comb generator (blue line). **a** Reprinted with permission from Ref. [89]. Copyright 2018, Optica. **b** and **c** Reprinted with permission from Ref. [63]. Copyright 2019, American Physical Society. **d**–**f** Reprinted with permission from Ref. [175]. Copyright 2022, Springer Nature

Combining periodic poling LN (PPLN) waveguides with the intensified nonlinear interaction in microresonators has considerably improved the SHG efficiency [63, 120–122]. In 2018, Wolf et al. demonstrated QPM-based SHG in on-chip microresonators for the first time. The normalized conversion efficiency was measured to be 90%/W, and the microring is shown in Fig. 2a [89]. In 2019, Chen et al. realized ultra-efficient SHG in a QPM microring, whose loaded *Q*-factor was 3.7 × 10^5^, and the conversion efficiency reached 230,000%/W [74]. Moreover, a second-harmonic conversion efficiency of 250,000%/W was achieved in a PPLN microring in the same year, where QPM was realized by field-assisted domain engineering [70]. In 2020, by optimizing the *χ*^(2)^ photon-photon coupling strength toward single-photon nonlinearity in a PPLN microring, Lu et al. utilized the largest *χ*^(2)^ tensor element *d*_33_ and implemented a high-fidelity radial poling in the microring; a record conversion efficiency of 5,000,000%/W and a single-photon coupling rate of 1.2 MHz were demonstrated [76]. Benefiting from the PPLN microdisk with an ultrahigh *Q*-factor up to 1.2 × 10^8^, a normalized conversion efficiency of 470,000%/W was reached, presenting a maximum absolute conversion efficiency as high as 23% [40]. In 2021, Chen et al. realized an absolute second-harmonic conversion efficiency of (58 ± 3)% with (3.4 ± 0.1) mW pump power in a PPLN microring and the normalized conversion efficiency was measured to be (137,000 ± 8100)%/W. Assisted by an on-chip phase modulator to stabilize SHG in the high-pump-power region, the device can maintain stable SHG with a conversion efficiency around 50% for about 30 min via Pound–Drever–Hall locking to stabilize the frequency of a laser to a microresonator [123]. Besides microdisk and microring resonators, PhC cavities have also been studied extensively for SHG [124, 125]. In 2019, Li et al. demonstrated 2D LN PhC slab nanoresonators with a *Q*-factor up to 3.51 × 10^5^ at 1537 nm, allowing an orientation-dependent second-harmonic conversion efficiency of 0.078%/W. The small conversion efficiency was primarily due to the low *Q*-factor associated with the second-harmonic wave with significant radiation leakage into free space [125]. Yuan et al. experimentally demonstrated strongly enhanced SHG in 1D heterostructure cavities on TFLNOI [126]. Combining a guided-mode resonance with distributed Bragg reflectors, a high normalized conversion efficiency of 2.03 × 10^−5^ cm^2^/GW was exhibited at a pump intensity of 0.05 GW/cm^2^. A more detailed summary about SHG on TFLNOI can be found in Ref. [127].

SFG on TFLNOI has also been demonstrated on resonator-based devices [97, 115, 128, 129]. By pumping a resonator (*Q*-factor ~ 10^5^) with two individual lasers, a sum-frequency conversion efficiency of 0.014%/W was first reported in an on-chip LN microdisk [128]. Phase matching methods have also been applied in the SFG process to notably boost the efficiency of SFG. Ye et al. realized efficient SFG through two individual CW pumps with powers of several milliwatts, and the conversion efficiency was measured to be 0.222%/W through MPM [97]. Utilizing a fully optimized PPLN microring in the over-coupling state, ultra-efficient SFG was achieved on a chip. The quantum efficiency was observed to be (65 ± 3)% with (104 ± 4) μW pump power and only 3 × 10^5^ noise photons were created over the cavity life time [115]. Jiang et al. demonstrated flexible SFG in a high-*Q* LN PhC nanobeam resonator with a *Q*-factor of 5.42 × 10^4^ at 1504.7 nm; the conversion efficiency agreed well with the theoretical expectation [124].

Low-threshold frequency down-conversion process has been demonstrated so far in crystalline LN [130, 131]. Owing to the MPM mechanism, not only was a high SHG conversion efficiency achieved, DFG was also observed in LN microrings, indicating a conversion rate of about − 53 dB [66]. By numerical simulations, Yang and Wang proposed a hybrid LN/Si platform to achieve terahertz generation via the difference-frequency process [132], and the terahertz generation efficiency could be increased to 2.1%/W in a racetrack microresonator. Ultralow-threshold (30 μW) OPO based on PPLN microrings have recently been reported, the OPO wavelength can be widely tuned by changing the pump wavelength and temperature; an on-chip power conversion efficiency of 11% was measured with a pump power of 93 μW [133]. Based on a high-*Q* microdisk, broadband non-degenerated OPO has been observed through natural QPM and the threshold was as low as 19.6 μW [40]. Over the past 5 years, several high-performance SPDC devices based on LN microresonators have been demonstrated [61, 113, 134]. Using an on-chip PPLN microring, a high photon-pair generation rate of 2.71 MHz/μW was reported. Moreover, the maximal coincidence to accidental ratio (CAR) was ~ 15,000, and the minimal autocorrelation of 0.008 was achieved [113]. Xu et al. utilized an LN microdisk to generate photon pairs covering a broad bandwidth of 30 nm by BPM [134]. Meanwhile, the photon-pair generation rate reached 5.13 MHz/μW with CAR up to 804, and the autocorrelation was measured to be 0.0098 ± 0.0021.

In high-*Q* LN microresonators, high-order harmonics and wave mixing effects can be generated by cascading *χ*^(2)^ processes. For instance, third-harmonic generation (THG) can be demonstrated as a result of cascaded SHG and SFG [63, 88], and four-wave mixing can be realized through cascaded SHG and DFG [135–137]. Lin et al. demonstrated THG in a microdisk with the broadband natural QPM mechanism. Due to the utilization of the highest nonlinear coefficient *d*_33_ of LN, a THG normalized conversion efficiency of 1.05%/mW^2^ was estimated. Figure 2b shows a SEM image of the microdisk and Fig. 2c presents the spectrum comprising of the pump light, second-harmonic wave and third-harmonic wave [63]. Based on a microdisk with MPM, effective FWM has been experimentally realized by introducing a signal light into the microdisk, the FWM was generated via cascaded SHG and DFG [136]; the normalized FWM conversion efficiency was approximately 10^−4^. In addition, a mixture of nonlinear processes was observed in a microring, including stimulated Raman scattering, SHG and SFG [138].

The EO effect may be the most appealing property of LN. Based on the EO effect, various EO devices can be created on the microresonator platform [90, 109, 139–144], among which the EO modulator is the most widely used [145–148]. In the early stage of integrated modulators, an on-chip modulator was usually designed on hybrid platforms, such as hybrid Si and LN [149, 150]. The EO effect of LN was used to substitute for the plasma dispersion effect of Si. Due to the large *d*_33_ and the shorter response time, modulators fabricated on TFLNOI possess better performances in terms of tuning rate and bandwidth [151–154]. In 2015, Wang et al. demonstrated efficient EO modulation in a microdisk with a *Q*-factor up to 1.19 × 10^6^, and an effective resonance-frequency tuning rate of 3.0 GHz/V was achieved [155]. With on-chip in-plane microelectrodes and high-*Q* microdisks, a more compact device with a tuning efficiency of 3.41 pm/V was realized [99]. A tuning efficiency of ~ 38 pm/100 V with a tuning range of ~ 400 pm was observed after optimization of the electrode geometry [156]. A microdisk with air-bridge wiring was proposed and demonstrated in Z-cut LN; a high tuning efficiency of 29.2 pm/V and large free spectral range (FSR) of 17.1 nm were realized with delicately designed ring electrodes [157]. Compared with the use of microdisks, there exist many advantages to fabricate modulators based on microrings with flexible electrode design [141, 158]. In 2018, Wang et al. realized efficient and linear tuning in a micro-racetrack modulator. The EO efficiency was measured to be 7.0 pm/V with good linearity and no observable changes in the extinction ratio and linewidth of the resonance, and an EO bandwidth of 30 GHz with a data transmission rate as high as 40 Gb/s was achieved [151]. Feng et al. proposed and demonstrated an ultrahigh-linearity EO modulator based on a ring-assisted Mach–Zehnder interferometer (MZI) by engineering the coupling efficient *κ* [159]. Through pure coupling modulation in an MZI coupled with a microring, a high-performance EO modulator on TFLN has been demonstrated recently, exhibiting a low insertion loss of 0.15 dB with a footprint of 3.4 mm × 0.7 mm [160]. The device also presented a large bandwidth of 67 GHz, a low half-wave voltage of 1.75 V and a driverless data transmission rate up to 240 Gb/s. High-speed EO modulator has also been fabricated based on a PhC nanobeam resonator with a loaded *Q*-factor of 1.34 × 10^5^, exhibiting a tuning efficiency of 16.0 pm/V and a 3-dB bandwidth of 17.5 GHz [153]. Compared with Mach–Zehnder modulators, resonator-based EO modulators can effectively reduce the required interaction length to achieve more compact devices and lower power consumption. However, the modulation bandwidth is limited by the RC time constant and the photon lifetime of the resonator [161]. To improve the bandwidth of a resonator-based modulator, an ultra-compact EO modulator based on a 2 × 2 Fabry–Perot cavity has been demonstrated on X-cut LN recently [162]. Owing to the reduced photon lifetime due to the small *Q*-factor, the modulation bandwidth of the device was measured to be over 110 GHz.

One of the most common methods for optical frequency comb (OFC) generation is based on the EO effect [163–167]. Through EO modulation, a series of coherent and equidistant frequency components are generated on both sides of the pump light and presented as an OFC [168, 169]. Optical microresonators are often regarded as ideal platforms for achieving efficient broadband EO combs, in which the optical frequency and modulation frequency are multiples of the resonator FSR [170–173]. In 2019, Zhang et al. demonstrated a broadband EO comb in an X-cut LN microring with a *Q*-factor of ~ 10^6^. When the modulation frequency and modulation index were 10.453 GHz and 1.2π, respectively, the EO comb was obtained, featuring a bandwidth exceeding 80 nm and having more than 900 comb lines with a slope of 1 dB/nm [174]. Moreover, efficient tuning of the modulation frequency from 10 Hz to over 100 MHz was realized; and comb bandwidth over a full octave was allowed by engineering the LN waveguide dispersion. Recently, a high-efficiency and broadband EO comb generator has been demonstrated based on coupled microresonators (Fig. 2d) [175], and the record pump-to-comb conversion efficiency and comb span of about 30% and 132 nm, respectively, were achieved with a microwave signal of 30.925 GHz. To overcome the trade-off between the comb span and the conversion efficiency, a tight binding model was proposed to design the coupled-resonator EO comb generator under the generalized critical coupling condition. Furthermore, the on-chip device can act as a femtosecond pulse source with duration of 336 fs. Figure 2e exhibits the relation between the comb power and the pump power, and Fig. 2f shows the measured EO comb spectrum. Through harnessing *χ*^(2)^ and *χ*^(3)^ nonlinearities on a single chip, a hybrid Kerr soliton and EO comb platform based on TFLNOI was demonstrated [176]. Direct electronic detection and feedback control of the soliton repetition rate were realized, when a high-repetition-rate Kerr soliton mode spacing was divided by the low-repetition-rate EO comb lines in the same waveguide.

### AlN

AlN features the highest bandgaps (6.2 eV) among all known *χ*^(2)^ nonlinear materials [177–179], and thus it provides suppression of two-photon absorption and a wide transparency window from UV to mid-infrared. This enables high-performance devices that can operate in a wide wavelength range. In addition, AlN possesses second-order nonlinearity, with an electro-optic coefficient *r*_33_ = 1 pm/V [41] and a second-order nonlinear susceptibility *χ*^(2)^ = 4.7 pm/V [42]. Furthermore, AlN has a superior thermal conductivity (*Ҡ*_AlN_ = 285 W/(m·K)) and a small thermo-optic coefficient (d*n*_AlN_/d*T* = 4.26 × 10^−5^/K) [180]. Hence, AlN-based photonic devices are able to handle high optical power. These properties make AlN an excellent platform for exploring various on-chip nonlinear optical processes. Previous successes in AlN-based nonlinear photonics came from polycrystalline AlN thin film, which is usually sputter‐deposited on a target substrate. Since the refractive index of AlN is around 2.1 at 1550 nm [181], SiO_2_ on Si wafers are typically chosen for the ease of fabrication and obtaining high index contrast. However, due to the scattering and absorption at the grain boundaries, *Q*-factors of sputtered AlN microresonators are limited to bellow the order of 10^5^ [182]. In contrast, single-crystalline AlN exhibits superior optical properties due to the improved crystalline quality and reduced grain size. High-quality AlN film is generally grown on sapphire substrate through metal-organic chemical vapor deposition (MOCVD) [183] or molecular beam epitaxy (MBE) [184]. By optimizing the fabrication process, the highest obtained intrinsic *Q*-factor of an AlN microring resonator is 3.7 × 10^6^ [43].

**Fig. 3:**
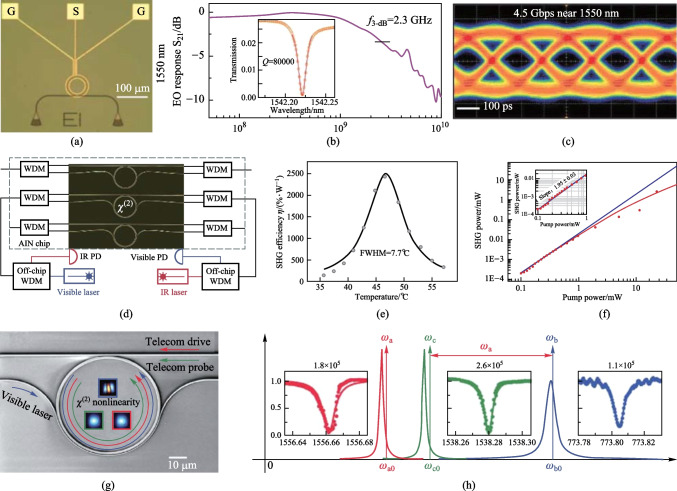
**a** Micrograph image of an AlN-based microring modulator with electrodes on top. **b** Frequency response of electro-optic modulation at telecom wavelengths. **c** Clear eye diagram under modulation speed of 4.5 Gbps near 1550 nm. **d** SHG experimental setup. An AlN microring resonator is coupled by two individual waveguides. **e** Temperature dependence of the SHG efficiency, with an optimized efficiency of 2500%/W achieved around 46 °C. **f** Pump power dependence of the SHG power on pump power. **g** SEM image of an AlN microring resonator. **h** Schematics of the optical resonance modes and the corresponding transmission spectra. **a**–**c** Reprinted with permission from Ref. [186]. Copyright 2012, American Chemical Society. **d**–**f** Reprinted with permission from Ref. [57]. Copyright 2016, Optica. **g**, **h** Reprinted with permission from Ref. [191]. Copyright 2016, American Physical Society

Due to low optical loss and relatively large EO coefficient, AlN can be used to achieve electro-optic devices [185]. In 2012, Xiong et al. demonstrated an AlN-based microring modulator [186]. As shown in Fig. 3a, a set of ground-signal-ground electrodes was placed above an oxide layer, while two grating couplers were used to couple light. The measured 3-dB bandwidth was 2.3 GHz in the telecom C-band for *Q*-factor of 8 × 10^4^ (Fig. 3b). The modulator supported a modulation speed of 4.5 Gb/s at 1550 nm (Fig. 3c), and it could also operate at visible wavelengths. However, the maximum electric field created by a voltage applied on the electrodes cannot be utilized due to the co-planer electrode structure. To increase the electric-optic overlap, Zhu and Lo proposed a parallel plate capacitor structure with the electrodes placed at the top and the bottom of the AlN waveguide [187]. In this way, the modulation efficiency was up to 320 V⋅cm for TM mode. Furthermore, the coherent conversion between microwave and optical signals was enabled based on the EO effect [188]. Planar superconducting resonators were integrated with an AlN microring on the same chip to meet the triple-resonance condition. An electromagnetically induced transparency effect was observed and an internal conversion efficiency of (25.9 ± 0.3)% was achieved.

Beyond the EO effect, efficient frequency conversions have been demonstrated on the AlN platform. In 2012, Pernice et al. reported SHG with a conversion efficiency of − 46 dB in AlN microring resonators [42]. The phase matching condition was fulfilled between the fifth-order mode at 775 nm and the fundamental mode at 1550 nm. In the follow-up work by the same group, efficient SHG was demonstrated in a dual-resonant AlN microring [57]. As shown in Fig. 3d, the top bus waveguide was used to couple the pump TM_0_ mode, while the bottom wrap-around waveguide was used to couple the second-harmonic TM_2_ mode. By optimizing the *Q*-factors, independently engineering the coupling conditions, and perfectly fulfilling the phase matching condition, a conversion efficiency up to 2500%/W can be realized (Fig. 3e). The SHG power was 3.3 mW with a saturated power conversion efficiency of 12% (Fig. 3f). Furthermore, Surya et al. demonstrated that by varying the cross-sectional dimension of the waveguide and radius of the AlN microring, as well as controlling the chip temperature, efficient SHG can be achieved at a specific wavelength [189]. Although several approaches have been implemented for device optimization, the conversion efficiency is ultimately limited by the intrinsic *Q*-factor. In 2018, Bruch et al. showed a record high SHG conversion efficiency of 17,000%/W to date on a single-crystalline AlN microring resonator [190]. Such a high efficiency, compared to that for previous polycrystalline AlN devices, is attributed to the greatly increased *Q*-factors at both infrared and near-visible bands.

Apart from SHG, another three-wave mixing processes have also been extensively investigated. In 2016, Guo et al. reported the strong coupling between telecom and visible optical modes on a triply-resonant AlN microresonator (Fig. 3g) [191]. As shown in Fig. 3h, when mode *a* is driven by a telecom-band laser, photons in modes *b* and *c* can be interconverted through SFG/DFG processes. By using a similar device, a parametric down-conversion photon pair source has been demonstrated by the same group [192]. The generated photon pairs exhibited high brightness and nearly ideal purity, showing the potential for quantum information processing. Later in 2019, Bruch et al. demonstrated the first experimental observation of low-threshold OPO in an AlN microresonator [193]. By optimizing the MPM and dual-resonance conditions, a high conversion efficiency of 17% as well as a broad tuning range were achieved. Recently, Wang et al. reported efficient frequency conversion via a degenerate sum-frequency process, with the maximum conversion efficiency up to 42% [194]. Since both driving and signal lights are at near resonance with the same telecom mode, the phase matching condition becomes more flexible. Furthermore, they observed that cascaded *χ*^(2)^ and *χ*^(3)^ effects can amplify the converted signal.

Additionally, *χ*^(2)^ processes play a considerable role in optical microcomb generation [195–197]. Jung et al. demonstrated efficient frequency doubling of an external comb through the combination of SHG and SFG [198]. Also, enhanced *χ*^(2)^ effects can participate in the Kerr microcomb generation process and achieve a near-visible frequency comb [199–202]. Further, Bruch et al. reported Pockels microcomb solitons driven by three-wave mixing in an AlN microresonator [203]. When *χ*^(2)^ nonlinearity is sufficiently strong, cascaded OPO and SHG/SFG enable the generation of quadratic solitons in the near-infrared band by pumping the device in the near-visible band. Compared with typical Kerr solitons, the Pockels solitons feature low comb threshold and high pump-to-soliton conversion efficiency. Moreover, cascaded nonlinear processes have been used for achieving new functional devices. In 2018, Guo et al. demonstrated all-optical control of linear and nonlinear energy transfer by combining *χ*^(2)^ and *χ*^(3)^ processes [204]. Such a control was achieved by coherently coupling the target mode to a high-loss visible mode, which can prevent the photon emissions into the target mode. Cascaded *χ*^(2)^ processes are equivalent to an effective *χ*^(3)^ process. Cui et al. realized in situ control of effective Kerr nonlinearity through its interplay with the cascaded Pockels processes [205]. More recently, Wang et al. reported a synthetic five-wave mixing process (*χ*^(4)^) by incorporating the *χ*^(2)^ and *χ*^(3)^ processes in a single microresonator [206]. This process is confirmed by the quantum entanglement between visible and telecom photons. Abundant nonlinear effects and multiple available optical modes make AlN-based microresonators an ideal platform for exploiting quantum photonic devices.

### SiC

SiC is a well-known CMOS-compatible material, and has attracted significant attention due to its superior properties. It exhibits a high refractive index (~ 2.6 at 1550 nm), a wide transparent window (0.37–5.6 μm) [45], and a relatively large second-order nonlinearity [207]. Furthermore, it hosts a variety of optically addressable defects with long spin coherence time, so that advanced quantum devices can be realized [208–211]. As a semiconducting material with abundant polytypes, the most common studied polytypes of SiC include 3C-, 4H- and 6H-SiC. In the last decade, several techniques have been developed to fabricate SiC thin film on insulator (SiCOI) [212–214]. By optimizing the preparation and etching processes, high-performance photonic devices have been demonstrated. So far, the highest *Q*-factor of 3C-SiC microring resonators is 2 × 10^5^, which is limited by the intrinsic loss due to lattice mismatch [215]. On the contrary, 4H-SiC has a higher crystal quality and the microcavity *Q*-factor on this platform can reach 7 × 10^6^ [46]. High-*Q* SiC microresonators have been used to investigate various nonlinear processes [216].

**Fig. 4:**
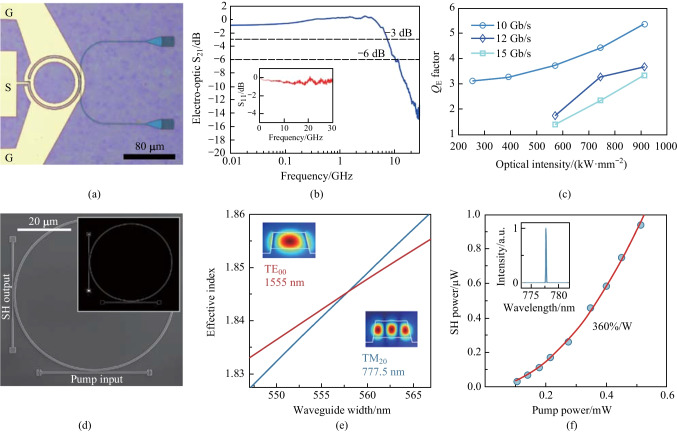
**a** Micrograph image of a 3C-SiC microring modulator. **b** Modulator bandwidth characterization; RF s-parameter characterization featuring a − 3-dB bandwidth of 7.1 GHz. **c** Eye diagram quality factors (*Q*_E_) as a function of the optical intensity. **d** SEM image of a 4H-SiC microring resonator. **e** Calculation of the phase-matching condition for the 1555-nm TE_00_ and 777.5-nm TM_20_ modes. **f** Dependence of the second-harmonic (SH) power on the pump power. A quadratic fit reveals a conversion efficiency of 360%/W. **a**–**c** Reprinted with permission from Ref. [44]. Copyright 2022, Springer Nature. **d**–**f** Reprinted with permission from Ref. [224]. Copyright 2020, Springer Nature

Despite the Pockels effect in bulk 3C-SiC was discovered more than three decades ago [217], integrated SiC EO modulators were not achieved until recent years [218, 219]. In 2021, Fan et al. reported the first experimental demonstration of integrated EO phase-shifter based on 3C-SiC racetrack microresonators [220]. This phase shifter supported a voltage-length product (*V*_π_·*L*_π_) of 118 V·cm, corresponding to an EO coefficient of 2.6 pm/V. In the meanwhile, a study by Powell et al. demonstrated a CMOS-compatible EO modulator based on a 3C-SiC microring resonator [44]. As shown in Fig. 4a, a pair of ground electrodes was placed next to the sides of the waveguide and a signal electrode was above the waveguide. The modulator showed a 3-dB bandwidth of 7.1 GHz (Fig. 4b). Moreover, it supported a transmission rate up to 15 Gbit/s and was able to operate at a high optical intensity of up to 913 kW/mm^2^ without degrading the signal (Fig. 4c). Thus far, the implementation of EO modulators is mainly based on 3C-SiC, and further exploration based on 4H-SiC platform is still needed.

Additionally, SHG has been explored in SiC microresonators. In 2014, Yamada et al. demonstrated SHG in a SiC-based PhC nanocavity by using a pulsed laser [221]. The conversion efficiency was estimated to be 0.15 W^–1^ with an average input power of 0.17 mW. By fabricating PhC nanocavities on 4H-SiC slabs, the same group achieved a higher conversion efficiency of 1900%/W [222]. Furthermore, they found that the nanometer-scale random imperfections introduced during fabrication caused a large uncertainty in the estimation of the SHG efficiency [223]. In 2020, Lukin et al. demonstrated efficient SHG in high-*Q* microring resonators based on the 4H-SiC platform (Fig. 4d) [224]. As shown in Fig. 4e, in order to utilize the *d*_31_ nonlinear coefficient, MPM was achieved between the fundamental quasi-TE mode (TE_00_) at 1555 nm and a quasi-TM mode (TM_20_) at 777.5 nm. The SHG efficiency was measured to be 360%/W (Fig. 4f). Moreover, a high-quality quantum emitter was also demonstrated in this work. In 2021, Wang et al. reported SHG with a conversion efficiency of 3.91%/W in a high-*Q* (6.75 × 10^6^) 4H-SiC microresonator [46].

### GaN

GaN is widely used in solid-state lighting as a wide-bandgap material [225–227]. Owing to the relatively high refractive index (*n*_o_ = 2.32 and *n*_e_ = 2.30) and nonlinear coefficient (*d*_36_ = − 4.6 pm/V and *n*_2_ = 1.4 × 10^−18^ m^2^/W), GaN is also a promising platform for integrated nonlinear photonics [228]. Moreover, the ultrawide transparency range from 365 nm to 13.6 μm provides the potential of generating wavelengths in the far-IR or near-UV by DFG or SFG on the GaN platform [229]. Recently, the fabrication process of GaN waveguides has been greatly improved. The propagation loss of GaN waveguides on an AlN buffer layer can be reduced to 0.65 dB/cm at 1.55 μm and 1 dB/cm at 1.56 μm [230, 231]; the GaN-on-sapphire waveguide loss can be as low as 2 dB/cm at 700 nm and 0.17 dB/cm at 1.55 μm [36, 192]. High-*Q* GaN-based microresonators have been demonstrated [232, 233], and a microring exhibiting an intrinsic *Q*-factor of 2.5 × 10^6^ was reported recently [36].

**Fig. 5:**
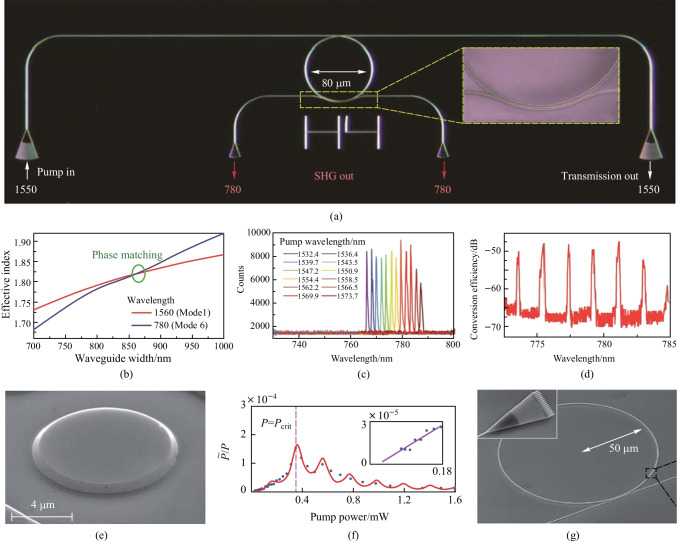
**a** Optical micrograph of GaN microring circuit for SHG; the inset shows a false-color SEM of the coupling region between the waveguide and the microring. **b** Calculation of the phase-matching condition between the fundamental mode at 1560 nm and the 6th order mode at 780 nm. **c** Tuning of SHG from 766 to 788 nm by aligning the pump light to different resonant wavelengths. **d** SH conversion efficiency as the pump wavelength continuously tuned from 1540 to 1568 nm. **e** SEM image of a GaP microdisk before being undercut. **f** Absolute efficiency of SHG when the pump is on-resonance; the inset shows the low P data and the linear fit. **g** SEM image of a GaP microring for *χ*^(2)^ and *χ*^(3)^ nonlinear photonics; the inset shows the grating coupler. **a**–**d** Reprinted with permission from Ref. [64]. Copyright 2011, Optica. **e**, **f** Reprinted with permission from Ref. [242]. Copyright 2016, American Institute of Physics. **g** Reprinted with permission from Ref. [37]. Copyright 2020, Springer Nature

In 2011, an integrated GaN microring on silicon was designed and demonstrated for strong SHG [64], as illustrated in Fig. 5a. By optimizing the waveguide structure, the phase matching condition was fulfilled and the second-harmonic wave at 780 nm was generated, as shown in Fig. 5b. As Fig. 5c presents, by aligning the pump light to different resonant wavelengths, the SHG can be tuned from 766 to 788 nm, demonstrating achievable broadband SHG on a chip. And the highest conversion efficiency of − 45 dB was measured under 120-mW input power (Fig. 5d). In 2017, a GaN slab PhC cavity with a *Q*-factor of 4.4 × 10^4^ was fabricated on silicon substrate for SHG, and a normalized conversion efficiency of 0.24%/W was reached [234]. Not only that, simultaneous SHG and THG were also observed in this PhC cavity. It should be noted that, there are only a few research reports on the second-order nonlinear processes in GaN microresonators, since integrated GaN nonlinear photonics is still in an initial development stage [235].

### GaP

GaP is an important and widely used material for fabrication of green light emitting diodes, with a bandgap of 2.26 eV. GaP is recognized as a potential material platform for integrated nonlinear photonics due to its intrinsic properties, including a large refractive index (*n* = 3.05 in the C- and L-bands), a high nonlinear coefficient (*d*_36_ = 159 pm/V at 852 nm) and a large transparency range (from 500 nm to 11 μm) [236, 237]. Furthermore, the minimal lattice mismatch between GaP and Si allows for heterogenous integration on the GaP platform, and GaP on SiO_2_ has been successfully fabricated through the wafer bonding process [238]. High-*Q* microrings have also been demonstrated on the GaP platform, featuring a *Q*-factor of 2.5 × 10^5^ and a propagation loss as low as 1.2 dB/cm [37]. Recently, a GaP PhC cavity with a *Q*-factor up to 1.8 × 10^6^ has been reported for microwave-to-optical frequency conversion [239].

In 2009, Rivoire et al. demonstrated efficient SHG with an input power of nanowatts coupled into a PhC cavity, and the normalized conversion efficiency was measured to be 430%/W [240]. In 2010, a doubly resonant PhC cavity with a *Q*-factor of 3800 was proposed and utilized to realize SFG on the GaP platform, and the sum frequency can be tuned by changing the cavity resonances [241]. In 2016, resonant SHG from 1550 to 775 nm with a normalized conversion efficiency beyond 38%/W was demonstrated in a GaP microdisk, and the double resonance condition was satisfied through temperature tuning. The GaP microdisk and the curve of SHG absolute efficiency are respectively shown in Fig. 5e, f [242]. Utilizing an integrated GaP-on-oxide microring, featuring a *Q*-factor around 10^4^, Logan et al. realized a normalized second-harmonic conversion efficiency of 400%/W via a QPM method [243]. In 2020, combining *χ*^(2)^ and *χ*^(3)^ nonlinearity in a microring with a radius of 50 um and a *Q*-factor of 2.5 × 10^5^, Kerr frequency combs and frequency-doubled combs were observed simultaneously. The frequency-doubled comb in the visible wavelength region was generated from the Kerr frequency comb at the C-band through *χ*^(2)^ nonlinearity, and the device is illustrated in Fig. 5g [37]. Based on GaP microresonators with high *Q*-factors and small mode volumes, the efficiency of *χ*^(2)^ nonlinearity can be improved further.

### AlGaAs

**Fig. 6:**
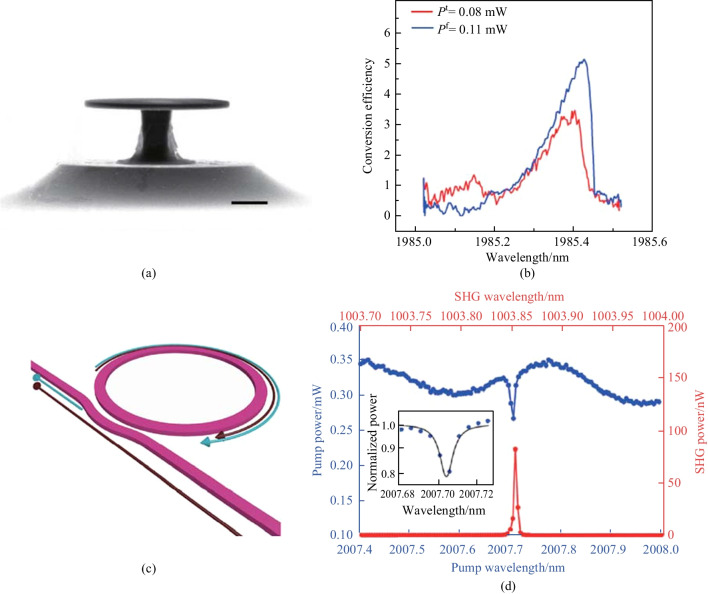
**a** SEM image of a QPM microdisk. **b** Second-harmonic external conversion efficiency as a function of the fundamental pump wavelength (at two fundamental powers, represented by the red and blue lines, respectively). **c** Schematic structure of a microring with a pulley coupler for efficient SHG. **d** Pump and second-harmonic spectra of the microring; the inset shows the resonance and fitting curve. **a**, **b** Reprinted with permission from Ref. [260]. Copyright 2020, Springer Nature. **c**, **d** Reprinted with permission from Ref. [263]. Copyright 2019, American Institute of Physics

Al_*x*_Ga_1_ _−_ _*x*_As (0 ≤ *x* ≤ 1), where the material concentrations of Al and Ga can be adjusted because of the fairly close lattice constants of GaAs and AlAs, is the most widely used III-V semiconductor in integrated photonics [244, 245]. (Al)GaAs presents a high refractive index (*n* = 3.4 at 1550 nm), a wide transparency window (from 870 nm to 19 μm), as well as strong *χ*^(2)^ (*d*_36_ = 119 pm/V) and *χ*^(3)^ (*n*_2_ = 2.6 × 10^−13^ cm^2^/V) nonlinearities [53]. High-*Q* microresonators have been demonstrated on the AlGaAs platform [52, 246–252], and an enormous amount of research has gone into *χ*^(2)^ nonlinear processes theoretically [253–256] and experimentally [257–259]. In 2013, Mariani et al. designed and fabricated an Al_0.4_Ga_0.6_As microdisk on a GaAs pedestal for SHG, and an absolute conversion efficiency of 4% was estimated [257]. Based on the same microdisk, SHG was also demonstrated in the telecom band, and a conversion efficiency of 0.07%/W was reached [258]. In 2014, it is proposed to use the $\overline{4}$ crystal symmetry in GaAs to enable the fabrication of QPM microdisks, and a GaAs microdisk with a radius of 2.6 μm and a thickness of 160 nm was fabricated to realize efficient SHG, as shown in Fig. 6a. The normalized conversion efficiency was measured to be 5%/W; when accounting for the fiber-cavity scattering, the external conversion efficiency can be 300%/W. Figure 6b illustrates the second-harmonic external conversion efficiency as a function of the fundamental pump wavelength [260]. A crossed beam GaAs PhC cavity suitable for nonlinear frequency conversion was also proposed and demonstrated, SFG and SHG processes were observed in this nanocavity [261]. The SPDC process has also been demonstrated in a suspended AlGaAs microdisk, and a bi-photon generation rate of 1.2 kHz/μW was measured under the QPM condition. A conversion efficiency of 5%/W for SHG was also estimated for the same device [262].

Though GaAs microresonators with *Q*-factor up to 6 × 10^6^ have been demonstrated on AlGaAs pedestal [52], the AlGaAs-on-insulator platform is considered as the compatible and suitable platform for nonlinear PICs [264, 265]. Based on this platform, high-index-contrast, high-material-quality waveguides can be fabricated in a mature and scalable way [266]. In 2019, Chang et al. first demonstrated efficient SHG in a microring fabricated on the AlGaAs-on-insulator platform [263], and the microring exhibited a high nonlinear optical coefficient and an intrinsic *Q*-factor exceeding 2.6 × 10^5^, as illustrated in Fig. 6c. Under 61-μW pump power coupled into the microcavity at the wavelength of 2 μm, an absolute internal conversion efficiency of 4% was realized; an internal normalized efficiency around 65,000%/W was calculated. Furthermore, an external conversion efficiency beyond 100,000%/W has been predicted for the GaAs microresonators, and the pump and SHG spectra of the microring is shown in Fig. 6d. The generation of *χ*^(2)^ OFC on the AlGaAs platform was also theoretically predicted [267]. A more comprehensive and detailed review about AlGaAs nonlinear integrated photonics can be found in Ref. [38].

## Conclusion and outlook

In this review, we have presented an overview of *χ*^(2)^ nonlinear photonics in integrated microresonators over the past two decades. *χ*^(2)^ nonlinear effects offer numerous ways to modulate and generate coherent light. Recent development of novel material platforms for integrated microresonators provides appealing pathways to explore these effects in a compact and efficient manner. Despite the tremendous progress, there still exist several directions that deserve to be given more attention in the future.

On the one hand, various material platforms have different advantages. For instance, benefiting from large *r*_33_, LN is preferred for ultrafast EO modulators; semiconductor materials with direct bandgaps, such as AlGaAs and GaN, can serve as light sources; SiC exhibits exceptional superiority in the quantum regime owing to the spin defect structure. However, it is unrealistic for a specific material to meet all the performance requirements for different applications. Fortunately, through heterogeneous integration, more components can be incorporated on the same chip to build fully integrated PICs [268–273]. This paves the way to overcome the limitations of single-material systems [274, 275].

On the other hand, the frequency conversion efficiency of *χ*^(2)^ nonlinearity can be further improved, which is the key factor in quantum communications and light-emitting devices [276, 277]. Generally, two major methods are applied to boost the conversion efficiency of χ^(2)^ nonlinearity: improving the *Q*-factor, and engineering the phase matching condition. By optimizing material preparation and device fabrication process, ultrahigh-*Q* microresonators can be demonstrated on more material platforms to enhance the interactions of light and matter, thus leading to a higher conversion efficiency. In addition, engineering the three-wave mixing phase matching is essential for achieving efficient frequency conversion. As mentioned in Sect. 2.2, the most frequently used MPM was achieved among different mode families, but the small mode overlap hinders the frequency conversion efficiency. While utilizing QPM, the phase matching condition can be fulfilled within the fundamental mode family, where the large spatial mode overlap can maximize the interaction efficiency. However, QPM is mainly exploited in LNOI-based microresonators, and it is expected that more extensive research on QPM should be developed based on several platforms such as GaP and AlGaAs. Furthermore, phase-matching-free nonlinear processes have been realized based on metasurface structures [278] and 2D materials [279], showing the potential to achieve a high conversion efficiency.

Finally, it is crucial to accelerate the translation from the bench-top demonstrations of *χ*^(2)^ nonlinearity to practical applications. Several outstanding applications based on *χ*^(2)^ nonlinearity have so far been demonstrated in several areas ranging from EO modulation to quantum light sources, although it is still challenging to build chip-scale and board-scale devices with low energy consumption and excellent stability in an industrial environment. It can be expected that more excellent achievements of *χ*^(2)^ nonlinear photonics in integrated microresonators will emerge in the near future.
